# PTK7 is a novel oncogenic target for esophageal squamous cell carcinoma

**DOI:** 10.1186/s12957-017-1172-x

**Published:** 2017-05-25

**Authors:** Kang Liu, Guiqin Song, Xuqian Zhang, Qiujiang Li, Yunxia Zhao, Yuchuan Zhou, Rong Xiong, Xin Hu, Zhirong Tang, Gang Feng

**Affiliations:** 1 Institute of Tissue Engineering and Stem Cells, The Second Clinical Medical College of North Sichuan Medical College, Nanchong Central Hospital, 637000 Nanchong, Sichuan Province People’s Republic of China; 2 Biotherapy Center, Nanchong Central Hospital, Nanchong, Sichuan People’s Republic of China; 3 Department of Biology, North Sichuan Medical College, Nanchong, Sichuan People’s Republic of China; 4 Department of Pathology, Nanchong Central Hospital, Nanchong, Sichuan Province China; 5 Clinical college of North Sichuan Medical College, Nanchong, Sichuan People’s Republic of China

**Keywords:** PTK7, Esophageal squamous cell carcinoma, Tumorigenesis, Metastasis

## Abstract

**Background:**

Overexpression of PTK7 has been found in multiple cancers and has been proposed to serve as a prognostic marker for intrahepatic cholangiocarcinoma. Its role in esophageal cancer, however, remains to be clarified. We hypothesize that PTK7 positively regulates tumorigenesis of esophageal cancer.

**Methods:**

We examined PTK7 expression pattern in human esophageal squamous carcinoma by Oncomine expression analysis and by immunohistochemistry (IHC) staining. We knocked down *PTK7* in two esophageal squamous cell carcinoma cell lines, TE-5, and TE-9, by siRNA, and evaluated cell proliferation, apoptosis, and migration of*PTK7*-defective cells. Expressions of major apoptotic regulators and effectors were also determined by quantitative real-time PCR in *PTK7*-defective cells. We further overexpressed PTK7 in the cell to evaluate its effects on cell proliferation, apoptosis, and migration.

**Results:**

Both Oncomine expression and IHC analyses showed that PTK7 is overexpressed in clinical esophageal squamous cell carcinoma tumors. *PTK7* siRNA suppressed cell growth and promoted apoptosis of TE-5 and TE-9. *PTK7-*defective cells further displayed reduced cellular migration that was concomitant with upregulation of E-cadherin. Conversely, overexpression of PTK7 promotes cell proliferation and invasion, while apoptosis of the PTK7-overexpressing cells is repressed. Notably, major apoptotic regulators, such as p53 and caspases, are significantly upregulated in si*PTK7* cells.

**Conclusions:**

PTK7 plays an oncogenic role in tumorigenesis and metastasis of esophageal squamous carcinoma. PTK7 achieves its oncogenic function in esophageal squamous cell carcinoma partially through the negative regulation of apoptosis.

## Background

Protein tyrosine kinase-7 (PTK7) is a member of receptor protein tyrosine kinases (RTKs) and comprised of three domains: an extracellular immunoglobulin-like domain, a transmembrane domain, and an inert tyrosine kinase-like domain [1]. Since the conserved catalytic residues are absent from its kinase-like domain [2–4], PTK7 lacks functional catalytic tyrosine kinase activity like other RTKs, and hence is classified as a pseudokinase. Despite being a pseudokinase, PTK7 is considered as a potentially important prognostic marker for many cancers. Overexpression of PTK7 has been found in colon cancer, lung cancer, gastric cancer, acute myeloid leukemia, and intrahepatic cholangiocarcinoma [5–11]. Disruption of PTK7 expression can repress cell proliferation and can promote apoptosis in colon and liver cancers [11, 12]. More importantly, intrahepatic cholangiocarcinoma patients with low PTK7 levels had longer disease-free and overall survivals than those with high PTK7 levels [11], suggesting it may serve as a prognostic predictor. On the other hand, PTK7 is a versatile co-receptor that can form complexes with Wnt, plexin, or vascular endothelial growth factor receptor 1 (VEGFR1) to regulate morphogenetic movement, axon guidance, cell migration, and tube formation during angiogenesis and differentiation [13–17]. Notably, given that Wnt signaling plays significant roles in human cancer [18] and that a recent study has shown that knockdown of PTK7 can inhibit Wnt/β-catenin activity [2], it is conceivable that PTK7 may also be involved in cancer development and progression. Although dysregulation of PTK7 has been reported in some cancers, its role in tumorigenesis and oncogenic progression awaits further demonstration. Therefore, we become motivated to investigate the functions of PTK7 in esophageal squamous carcinomas in the current study.

Esophageal cancer is one of the commonly diagnosed cancers globally and is one of the leading causes of cancer-related deaths worldwide. Squamous cell carcinoma and adenocarcinoma are the two major types of esophageal cancer, each of which is associated with different risk factors. Prognosis of esophageal cancer patients is very poor, with the overall 5-year survival rate lingering below 15% in the USA [19]. Most patients die within 1 year of diagnosis, because the disease has already progressed to advanced stages by the time when first symptoms appear. Despite advancement in diagnosis and management of esophageal cancer, its prognosis is hardly improved. However, if the cancer could be diagnosed at early stages, the 5-year survival rate would be significantly improved to about 80%. Currently, treatment of esophageal cancer is still dictated by the clinical parameters, including the TNM (tumor, node, and metastases) stages and the histological tumor characteristics. However, some reports have suggested that tumor heterogeneity is associated with prognosis [20, 21]. Even similarly staged tumors may respond very differently to the same treatment. Therefore, it is imperative to discover new biomarkers to assist with stratification of patients for customized treatment and to minimize the unfavorable side effects or costs that are associated with the current standard chemotherapy or radiotherapy.

The goal of the current study is to investigate the role of PTK7 in the oncogenic progression of esophageal cancer. Specifically, we evaluated the expression of PTK7 in esophageal cancer by Oncomine expression analysis and validated the observation in clinical tumor samples by immunohistochemistry (IHC) staining of the PTK7 protein. We knocked down *PTK7* in two esophageal squamous carcinoma cell lines and measured proliferation and apoptosis of these *PTK7*-deficient cells. We further characterized migration and invasion of these *PTK7*-knockdown cells. The current data suggest that PTK7 plays an oncogenic role in the proliferation and metastasis of esophageal squamous carcinoma cells. Importantly, PTK7 achieve its oncogenic function in human esophageal squamous cell carcinoma partially through the attenuation of apoptosis. Given the fact that the esophageal cancer biomarkers are scarce for clinical use, our ongoing work on PTK7 has significant implications in the diagnosis and treatment of this cancer.

## Methods

### Cell culture

Human esophageal squamous carcinoma cell lines, TE-5, and TE-9, were purchased from RIKEN Cell Bank (Tsukuba, Japan). All cell lines were maintained in RPMI-1640 (ThermoFisher Scientific, USA) containing 10% fetal bovine serum in a humidified atmosphere of 5% CO2 and 95% air at 37 °C.

### RNA interference

*PTK7* small interference RNA (*PTK7* siRNA) was previously reported [11] and synthesized at Shanghai GenePharmaCo. The siRNA sequences are 5′-GGC AUG UCU UCA AUC UCU GCU AGG UGA-3′ and5′-ACC UAG CAG AGA UUG AAG ACA UGCC-3′, and the following scrambled siRNA was used as the control: 5′-GAGUUAAAGUCAAAGUGACTT-3′ and 5′-GUCACUUUGACUUUAACUCTT-3′. BLAST search was performed against the human genome database and the above sequence was confirmed to be *PTK7*-specific. TE-5 and TE-9 cell lines were transfected with *PTK7*-specific siRNA and scrambled negative siRNA at a final concentration of 50 nM with G-fectin transfection reagent (Genolution Pharmaceuticals, Seoul, Korea). The transfected cells were maintained in culture until 72 h and were collected for analysis.

### Proliferation assay

Cells (1 × 10^4^/well) were seeded in a 96-well plate for 12 h. At different time points (i.e., 0, 24, 48, 72 h), cell viability was evaluated using a MTT cell proliferation kit (Roche, Mannheim, Germany), based on reduction of a tetrazolium salt into a water-soluble formazan dye in a living cell. Absorbance at 450 nm at each time point was recorded for both targeted-knockdown (si*PTK7*) and knockdown control (siControl) cells.

### Analysis of apoptosis

Annexin V-FITC-propidium iodide (PI) double staining was applied to analyze apoptosis among the targeted knockdown or control knockdown cells. Cells were harvested, washed, and re-suspended by cold PBS at a density of 1 × 10^6^ cells/mL, followed by co-incubation with a mixture of PI (final concentration 100 μg/mL) and Annexin V-Alexa Fluor488 at room temperature for 15 min. Apoptosis was analyzed by flow cytometry with 494/518 nm set for Annexin V detection and 535/617 nm for PI.

### Cellular migration assay

Cellular migration assay was performed with transwell apparatus (Falcon 354480; BD Biosciences). *PTK7*-knockdown and scramble iRNA controls cells were harvested andre-suspended with serum-free DMEM medium at a concentration of1 × 10^5^ cells/mL. To the upper chamber, 100 μL of the cell suspension was added. To the lower chamber, 500 μL of DMEM medium containing 20% FBS was filled in. For negative control, medium containing 1% FBS was filled into the lower chamber. After incubation at 37 °C for 20 h, migrated cells at the bottom chamber were fixed by 4% paraformaldehyde and stained with 0.5% crystal violet. Five microscopic fields (magnification, ×100) were randomly photographed. Experiments were repeated three times for statistical analysis.

### Western blot

Whole cell lysates were separated on 12% SDS-polyacrylamide gels and were transferred to polyvinylidene difluoride (PVDF) membranes. Membranes were blocked in 5% non-fat milk in Tris-buffered saline and 0.1% Tween-20. PTK7 (Cat. 11926, Cell Signaling, USA), E-cadherin (Cat. ab1416, Abcam, USA), and β-actin (Cat. ab8226, Abcam, USA) antibodies were used in the western blot analysis.

### Immunohistochemistry (IHC)

Paraffin-embedded blocks of tumor or normal tissues were deparaffinized and rehydrated, before antigen retrieval was performed by microwaving in citrate-buffered solution (pH6.0). Goat serum (10%) was used for blocking. Sections were incubated with PTK7 antibodies overnight in a humidified container at 4 °C. A secondary antibody conjugated with horseradish peroxidase was then incubated with the sections for 1 h at room temperature. Finally, sections were stained and counterstained by 3, 3-diaminobenzidinetetrahy drochloride (DAB) and hematoxylin, respectively.

### Statistical analysis

Student’s tests were performed to analyze the in vitro data. A χ^2^test was performed to test the correlation of positive PTK7 staining with clinical tumor samples. A *P* value < 0.05 was considered significant. All tests were performed using the SPSS 17.0 software (SPSS, Chicago, IL, USA).

## Results

### PTK7 is upregulated in human esophageal squamous cell carcinoma

**Fig. 1:**
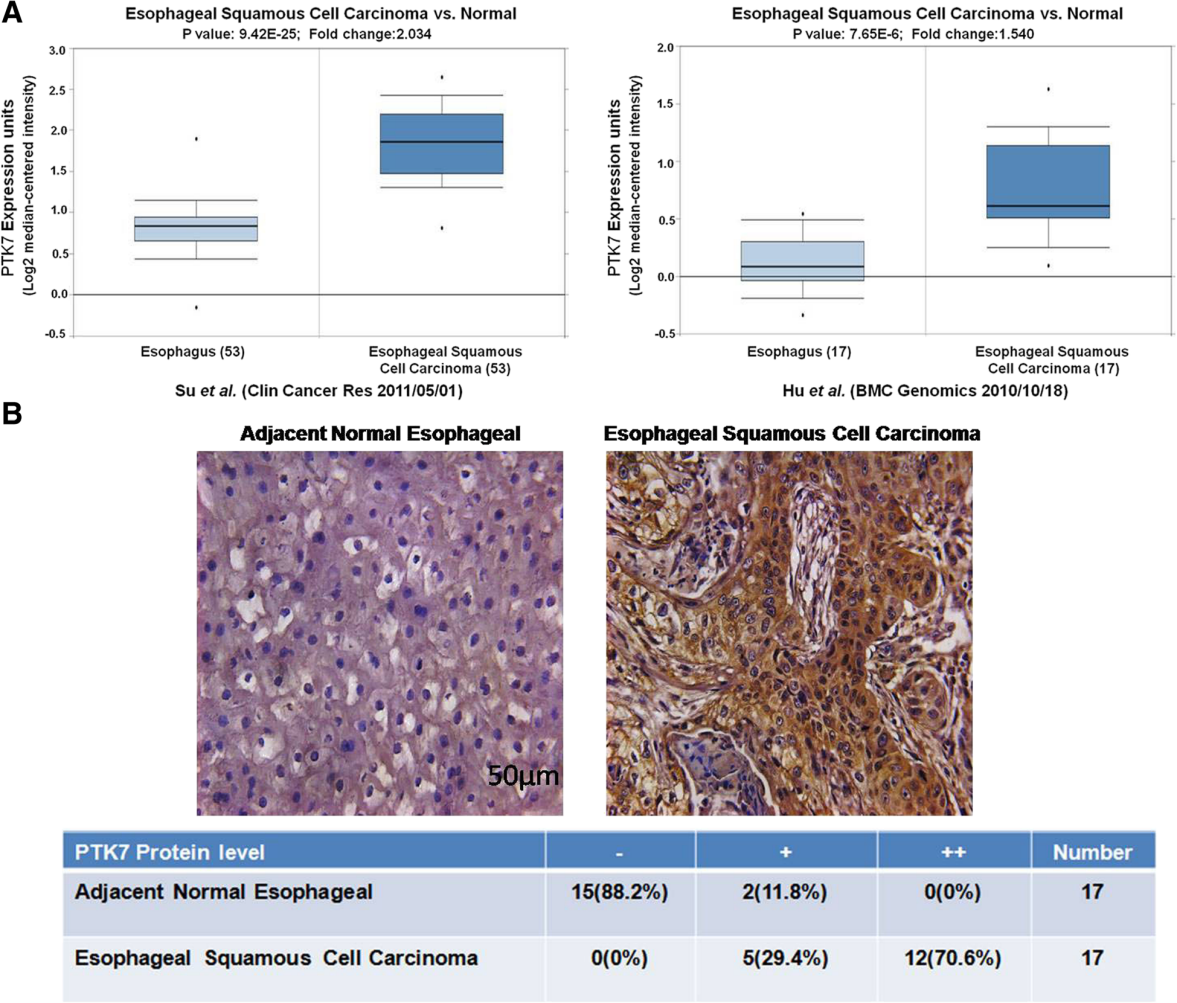
PTK7 is upregulated in esophageal squamous cell carcinoma tumors. **a** Oncomine expression analysis of PTK7 in human esophageal squamous cell carcinoma tumors vs. normal esophageal tissues. **b** IHC staining for PTK7 in clinical esophageal squamous cell carcinoma tumors and adjacent normal tissues. The table tabulates the statistics of the negative (−), positive (+), and strong staining (++) of PTK7 in the tumors and the normal tissues. A χ^2^test was performed to confirm the correlation of positive PTK7 staining with the tumor samples (χ^2^ = 166.318, df = 2, *p* < 0.001)

PTK7 has been reported to be upregulated in multiple cancers, including those of colon, lung, gastro, and leukemia. This prompted us to test if PTK7 is also regulated in esophageal squamous cell carcinoma. We performed Oncomine expression analysis for PTK7 based on the previously published research [22, 23]. Interestingly, in both studies, PTK7 is expressed 1.5-fold or higher in esophageal squamous cell carcinoma than in the normal esophageal tissues, and the difference is statistically significant (Fig. 1a). Consistently, IHC analysis showed markedly increased level of PTK7 in the clinical tumors samples of esophageal squamous cell carcinoma than the adjacent normal tissues, and strong staining is predominantly present in the cytoplasm of the disarrayed tumor cells, which is in agreement with its presumable subcellular localization (Fig. 1b). Furthermore, in the clinical tumor samples we examined, positive or strong positive staining of PTK7is correlated with most tumor samples but not with normal adjacent tissues (Fig. 1b, χ^2^test, *p* < 0.001), strongly arguing for an oncogenic role of PTK7 in the tumorigenesis of human esophageal squamous cell carcinoma.

### Knocking down *PTK7* inhibits cellular proliferation in vitro

**Fig. 2:**
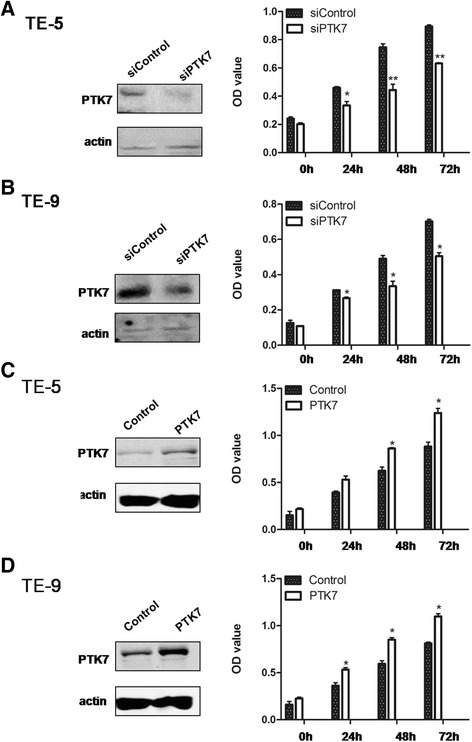
PTK7 positively regulates cell proliferation in esophageal squamous tumor cells. PTK7 level was knocked down by siRNA in human esophageal squamous carcinoma cell lines, TE-5 (**a**) and TE-9 (**b**). PTK7 western blot (*left panels*) was performed to confirm the siRNA results. Proliferation of the knockdown (si*PTK7*) and the control cells (siControl) was measured by MTT assays in TE-5 and TE-9 ( PTK7 was overexpressed in both TE-5 (**c**) and TE-9 (**d**) cells, cell proliferation was measured by MTT assays. (*p < 0.05, **p < 0.01, Student’s *t* test)

In light of overexpression of PTK7 in the clinical tumor samples of human esophageal squamous cell carcinoma, we further knocked down its level in two esophageal carcinoma cell lines, TE-5 and TE-9, by siRNA, of which the knockdown specificity has been confirmed [11]. Western analysis showed that PTK7 had been efficiently decreased. The MTT-based cellular proliferation assay for the knockdown (si*PTK7*) and the control cells (siControl) suggested that the growth of si*PTK7* was significantly slower than siControl (*p* < 0.01 for TE-5 and *p* < 0.05 for TE-9), indicating a promoting role of PTK7 in tumor cell proliferation (Fig. 2a, b).Supporting this notion, overexpression of PTK7 in both cell lines significantly promoted cell growth (Fig. 2c, d).

### Downregulation of *PTK7* promotes apoptosis

**Fig. 3:**
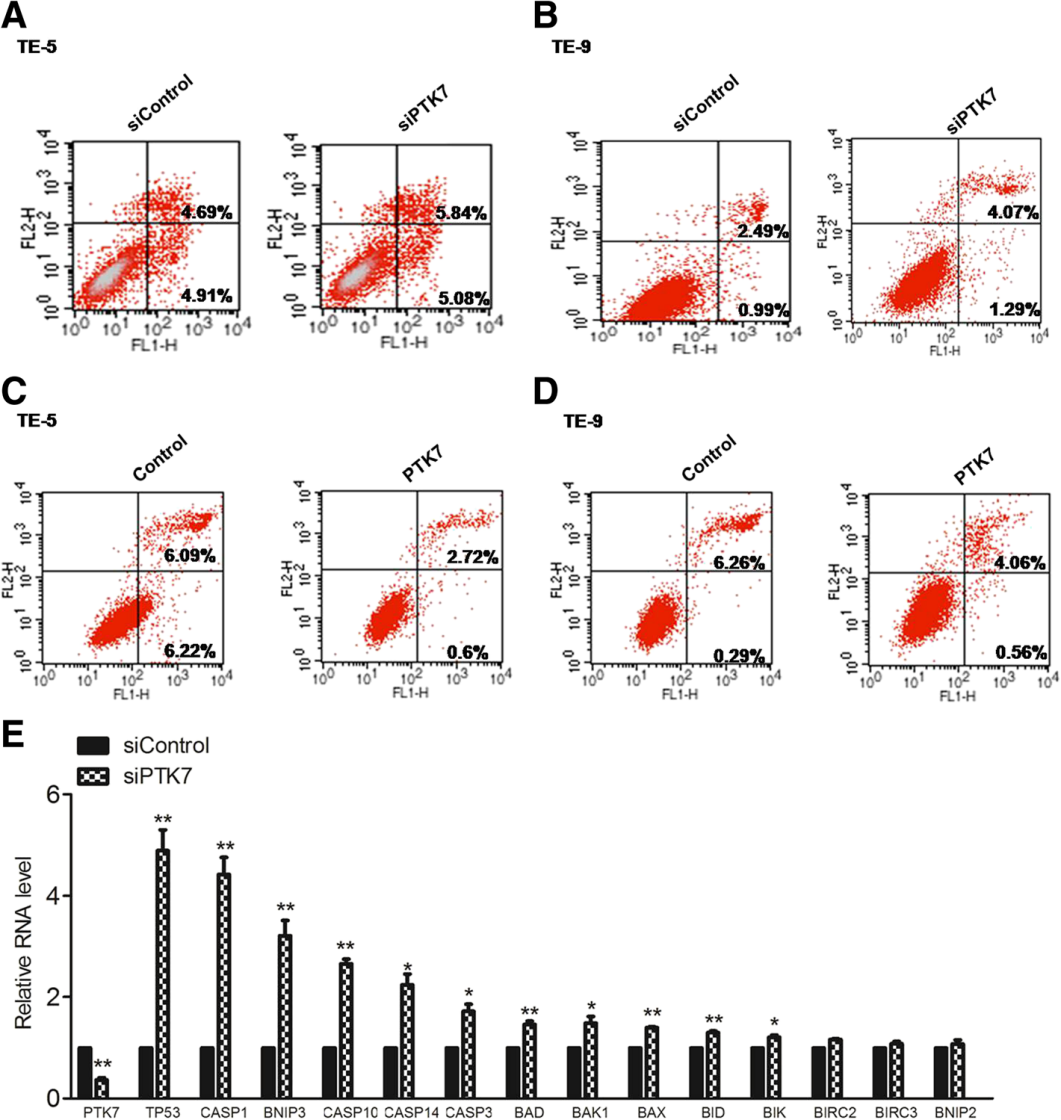
PTK7 negatively regulates cell apoptosis in esophageal squamous tumor cells. Apoptosis of the PTK7 knockdown cells and the control cells was evaluated by flow cytometry after double staining of Annexin V-FITC-propidium iodide for TE-5 (**a**) and TE-9 (**b**) cells. Apoptosis of PTK7-overexpressing and control cells was measured by flow cytometry after double staining of Annexin V-FITC and propidium iodidefor TE-5 (**c**) and TE-9 (**d**) cells. **e** Quantitative real-time PCR was performed for major apoptosis regulators, and the relative mRNA levels are presented for si*PTK7* vs. control cells. (*p < 0.05, **p < 0.01, Student’s *t* test)

To further test the role of PTK7 in cancer cell viability, we analyzed apoptosis of si*PTK7* and siControl cells by flow cytometry. We found that si*PTK7* cells had more apoptotic cells than siControl ones. Notably, in both cases of TE-5 and TE-9, si*PTK7* cells had increased populations of both early stage (Annexin V^+^/PI^−^) and late stage apoptotic (Annexin V^+^/PI^+^) cells (Fig. 3a, b). However, when PTK7 was overexpressed in both cell lines, the apoptotic populations were decreased instead, suggesting that PTK7 may positively regulate apoptosis (Fig. 3c, d). Substantiating this point, we found the major regulators and effectors of apoptosis, such as p53 and Caspases, were significantly upregulated in the si*PTK7* cells (Fig. 3e), suggesting PTK7 may play a major role in regulating apoptosis in esophageal squamous cell carcinoma.

### Knocking down *PTK7* decreases cellular migration in vitro

**Fig. 4:**
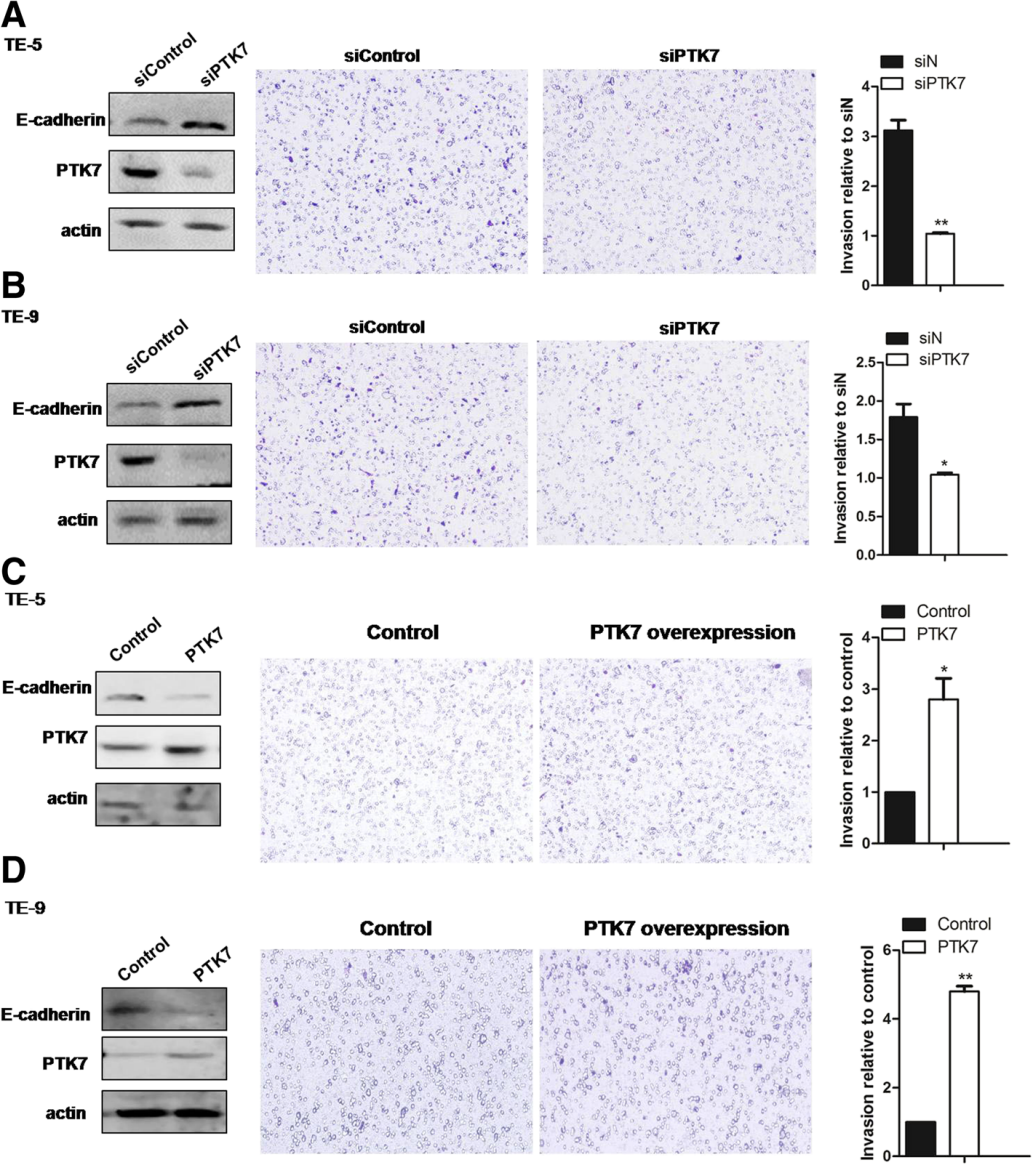
PTK7 positively cell invasion in esophageal squamous tumor cells. **a** and **b** western blots of PTK7 and E-cadherin were performed for the si*PTK7* and control cells in TE-5 (**a**, *left panel*) and TE-9 (**b**, *left panel*). Transwell assays were performed in TE-5 and TE-9 (**a** and **b**, *middle panels*). Quantification of the results shown in (**a** and **b**, *right panels*). **c** and **d** PTK7 was overexpressed in TE-5 and TE-9 cells. Western blots of PTK7 and E-cadherin (*left panels*), Transwell migration assays (*middle panels*), and the quantification of cell migration (*right panels*) of the PTK7-overexpressing and control cells are presented. (mean ± S.D., n = 3, *p < 0.05, **p < 0.01, Student’s *t* test)

To evaluate the role of PTK7 in tumor invasion, we compared the migration of si*PTK7*and siControl cells in vitro. Transwell migration assay showed that, in both cases of TE-5 and TE-9, migration of si*PTK7* cells was significantly reduced by 60% or more compared with siControl (*p* < 0.01) (Fig. 4a, b), suggesting a promoting role of *PTK7* in esophageal squamous carcinoma invasion. Interestingly, E-cadherin level was upregulated in si*PTK7* cells (Fig. 4a, b, western blots), further suggesting PTK7 may promote cell migration through downregulating epithelial-mesenchymal transition (EMT)-related pathways (see Discussion). On the contrary, overexpression of PTK7 downregulated E-cadherin and promoted cancer cell invasions (Fig. 4c, d).

## Discussion

In this study, we found that PTK7 plays an oncogenic role in esophageal squamous cell carcinoma. In light of Oncomine expression analysis and IHC staining results, we confirmed that PTK7 is overexpressed in clinical esophageal squamous cell carcinoma tumors. These observations lead us to test the role of PTK7 in tumor cell proliferation and migration, the hallmarks of tumorigenesis and metastasis. Indeed, disruption of PTK7 expression by siRNA rendered the tumor cells lose the proliferative advantages to the undisturbed counterparts. Importantly, more si*PTK7* cells undergo apoptosis than the controls, and major apoptotic regulators are upregulated in the *PTK7*-knockdown cells, suggesting PTK7 may be a major regulator of apoptosis in esophageal squamous cell carcinoma. More interestingly, disruption of PTK7 further slowed down cellular migration in vitro, during which the epithelial-mesenchymal transition marker gene E-cadherin was upregulated, suggesting PTK7 may promote metastasis of esophageal squamous cell carcinoma. To the best of our knowledge, this is the first report demonstrating the oncogenic role of PTK7by repressing apoptosis in esophageal squamous cell carcinoma.

In the current study, we found PTK7 plays a significant role in promoting tumor cell growth in esophageal squamous cell carcinoma cell lines. When PTK7 expression was disrupted by RNAi, the tumor cells proliferated slowly and became prone to apoptosis. PTK7 was initially identified in human colon carcinoma tissue and its derived cell lines, but it is not expressed in normal adult colon tissue [5]. Since it lacks conserved amino acid residues that are required for the receptor tyrosine kinase activity, its tumor-related functions must derive from other aspects than the kinase activity. Previous work has suggested that PTK7 may serve as a co-receptor for different receptors and selectively re-route signaling pathways to morphogenesis and angiogenesis during neuronal tube formation or planar cell polarity [13]. Complexed with Wnt, it also functions like a molecular switch to toggle between canonical and non-canonical Wnt signaling pathways [2]. Therefore, given the versatility of PTK7 and the critical role of Wnt in tumorigenesis, we speculate that the oncogenic role of PTK7 in esophageal squamous carcinoma can be ascribed to this “molecular switch”-like mechanism. Future work should be directed to determine the relationship between PTK7 and the Wnt signaling pathway. Particularly, the oncogenic characteristics of Wnt signaling should be examined for esophageal squamous carcinoma in the context of PTK7.

Interestingly*,* PTK7 may also play a role in metastasis of esophageal squamous carcinoma*. PTK7*-knockdown cells show noticeably decreased migration *in vitro* (Fig. 4). While further in vivo evidence awaits demonstration, we propose that PTK7 may positively regulate metastasis of esophageal squamous carcinoma. Furthermore, E-cadherin is upregulated in the *PTK7*-knockdown cells, showing a possibility that PTK7 positively regulates metastasis by promoting epithelial-to-mesenchymal transition (EMT). Loss of E-cadherin function or expression is a characteristic feature of cancer progression and metastasis [24], wherein cellular adhesion within a tissue is reduced and thus facilitates cellular motility and malignant invasion to adjacent tissues. Additionally, downregulation of E-cadherin by an otherwise different mechanism was reported to promote metastasis in esophageal cancer [25]. Therefore, based on the results obtained from the in vitro proliferation and invasion assays, we propose that PTK7 positively regulates tumorigenesis and metastasis of esophageal cancer.

## Conclusions

In summary, we have provided in vitro evidence that PTK7 promotes proliferation and migration of esophageal squamous cell carcinoma cells. Both Oncomine expression analysis and clinical tumor IHC confirmed that PTK7 is upregulated in esophageal tumors. The oncogenic role of PTK7 may be dependent on its negative regulation on apoptosis in the tumor cells. Therefore, PTK7 may be a promising therapeutic target in esophageal cancer, and its clinical implications should be further demonstrated by extensive and meticulous examination in clinical studies.

## Abbreviations

IHC: Immunohistochemistry
PTK7: Protein tyrosine kinase-7
RTKs: Receptor protein tyrosine kinases
VEGFR1: Vascular endothelial growth factor receptor 1
